# Empiric vs Preemptive Antifungal Strategy in High-Risk Neutropenic Patients on Fluconazole Prophylaxis: A Randomized Trial of the European Organization for Research and Treatment of Cancer

**DOI:** 10.1093/cid/ciac623

**Published:** 2022-07-30

**Authors:** Johan Maertens, Tom Lodewyck, J Peter Donnelly, Sylvain Chantepie, Christine Robin, Nicole Blijlevens, Pascal Turlure, Dominik Selleslag, Frédéric Baron, Mickael Aoun, Werner J Heinz, Hartmut Bertz, Zdeněk Ráčil, Bernard Vandercam, Lubos Drgona, Valerie Coiteux, Cristina Castilla Llorente, Cornelia Schaefer-Prokop, Marianne Paesmans, Lieveke Ameye, Liv Meert, Kin Jip Cheung, Deborah A Hepler, Jürgen Loeffler, Rosemary Barnes, Oscar Marchetti, Paul Verweij, Frederic Lamoth, Pierre-Yves Bochud, Michael Schwarzinger, Catherine Cordonnier, for the Infectious Diseases Group and the Acute Leukemia Group of the European Organization for Research and Treatment of Cancer

**Affiliations:** Department of Hematology, University Hospitals Leuven, Leuven, Belgium; Department of Hematology, Algemeen Ziekenhuis St Jan, Brugge, Belgium; Department of Hematology, Radboud University Medical Center, Nijmegen, The Netherlands; Department of Hematology, Caen University Hospital, Caen, France; Department of Hematology, Centre Hospitalier Universitaire Henri Mondor, Créteil, France; Department of Hematology, Radboud University Medical Center, Nijmegen, The Netherlands; Department of Hematology, Centre Hospitalier Universitaire Limoges, Limoges, France; Department of Hematology, Algemeen Ziekenhuis St Jan, Brugge, Belgium; Department of Hematology, University of Liège and University Hospital of Liège, Liège, Belgium; Department of Internal Medicine, Institut Jules Bordet, Brussels, Belgium; Department of Hematology/Oncology, Caritas Hospital, Bad Mergentheim, Germany; Department of Hematology/Oncology, Faculty of Medicine and Medical Centre, University of Freiburg, Freiburg, Germany; Department of Hematology, Masaryk University Brno and Institute of Hematology and Blood Transfusion, Prague, Czech Republic; Department of Internal Medicine/Infectious Diseases, Cliniques Universitaires St. Luc, Brussels, Belgium; Department of Oncohematology, Comenius University and National Cancer Institute, Bratislava, Slovakia; Service des maladies du sang, Centre Hospitalier Régional Universitaire Lille, Lille, France; Department of Hematology, Gustave Roussy Cancer Campus, Villejuif, France; Department of Hematology, Radboud University Medical Center, Nijmegen, The Netherlands; Department of Internal Medicine, Institut Jules Bordet, Brussels, Belgium; Department of Internal Medicine, Institut Jules Bordet, Brussels, Belgium; European Organisation for Research and Treatment of Cancer Headquarters, Brussels, Belgium; European Organisation for Research and Treatment of Cancer Headquarters, Brussels, Belgium; Merck & Co, Inc, Kenilworth, New Jersey, USA; Department of Internal Medicine II, Universitaetsklinikum, Würzburg, Germany; Department of Infection, Immunity and Biochemistry, Cardiff University, Cardiff, United Kingdom; Department of Medicine, Lausanne University Hospital, Lausanne, Switzerland; Department of Infectious Diseases, Ensemble Hospitalier de la Côte, Morges, Switzerland; Department of Hematology, Radboud University Medical Center, Nijmegen, The Netherlands; Department of Medicine, Lausanne University Hospital, Lausanne, Switzerland; Department of Medicine, Lausanne University Hospital, Lausanne, Switzerland; Translational Health Economics Network, Bordeaux University Hospital, Bordeaux, France; Department of Hematology, Centre Hospitalier Universitaire Henri Mondor, Créteil, France

**Keywords:** neutropenia, empiric, preemptive, antifungal, galactomannan

## Abstract

**Background:**

Empiric antifungal therapy is considered the standard of care for high-risk neutropenic patients with persistent fever. The impact of a preemptive, diagnostic-driven approach based on galactomannan screening and chest computed tomography scan on demand on survival and on the risk of invasive fungal disease (IFD) during the first weeks of high-risk neutropenia is unknown.

**Methods:**

Patients with acute myeloid leukemia (AML) or myelodysplastic syndrome (MDS) and allogeneic hematopoietic cell transplant recipients were randomly assigned to receive caspofungin empirically (arm A) or preemptively (arm B), while receiving fluconazole 400 mg daily prophylactically. The primary end point of this noninferiority study was overall survival (OS) 42 days after randomization.

**Results:**

Of 556 patients recruited, 549 were eligible: 275 in arm A and 274 in arm B. Eighty percent of the patients had AML or MDS requiring high-dose chemotherapy, and 93% of them were in the first induction phase. At day 42, the OS was not inferior in arm B (96.7%; 95% confidence interval [CI], 93.8%–98.3%) when compared with arm A (93.1%; 95% CI, 89.3%–95.5%). The rates of IFDs at day 84 were not significantly different, 7.7% (95% CI, 4.5%–10.8%) in arm B vs 6.6% (95% CI, 3.6%–9.5%) in arm A. The rate of patients who received caspofungin was significantly lower in arm B (27%) than in arm A (63%; *P* < .001).

**Conclusions:**

The preemptive antifungal strategy was safe for high-risk neutropenic patients given fluconazole as prophylaxis, halving the number of patients receiving antifungals without excess mortality or IFDs.

***Clinical Trials Registration.*** NCT01288378; EudraCT 2010-020814-27.

## Teaser

The initiation of antifungal therapy with caspofungin based on serum galactomannan screening and computed tomography scan when required in profound and prolonged neutropenic patients given fluconazole prophylaxis was found to be noninferior to a fever-driven empiric approach.

Prolonged and profound neutropenia, defined as <500 neutrophils/mm^3^ (<0.5 × 10^6^/L neutrophils) for at least 10 days, is a major risk factor for developing life-threatening invasive fungal diseases (IFDs) in patients with acute myeloid leukemia (AML) or myelodysplastic syndromes (MDS) receiving remission-induction or reinduction chemotherapy or undergoing myeloablative allogeneic hematopoietic cell transplantation (HCT). These patients benefit from antifungal agents when they present with neutropenic fever that has not been reduced after 3 to 7 days of broad-spectrum antibacterials. This empiric use of antifungals, which was explored in the 1980s when only culture and microscopy were available to diagnose IFD, became the standard of care [1, 2], supported by international guidelines [3, 4]. However, empiric use of the recommended antifungals liposomal amphotericin B and caspofungin [5, 6] most likely leads to overtreatment with increased toxicity and costs.

The availability of nonculture-based tests, such as the Platelia galactomannan enzyme-immunoassay (EIA) [7–10], and of computed tomography (CT) scanning [11, 12] has formed the basis of a so-called preemptive or diagnostic-driven approach. Instead of unexplained fever, abnormalities seen on a chest CT scan or mycologic test results trigger the start of antifungals [13]. Although several open-label and observational studies have reported promising results in terms of clinical outcomes and cost-effectiveness [14–25], there is still no consensus on the optimal design of a preemptive strategy.

Previously, both strategies were compared in the randomized PREVERT study [23]. However, there were too few patients with prolonged neutropenia to rule out the noninferiority of survival with the preemptive strategy. Therefore, the Infectious Diseases Group and the Acute Leukemia Group of the European Organization for Research and Treatment of Cancer (EORTC) initiated this new trial with overall survival as the primary end point.

## METHODS

### Study Design and Participants

The EORTC 65091-06093 study was an open-label, phase 3, randomized, parallel, multicenter, strategy trial comparing the efficacy and safety of a fever-driven antifungal approach (empiric, arm A) to a diagnostic-driven approach (preemptive, arm B) in neutropenic patients at high risk of developing IFD.

We recruited patients aged ≥18 years who were scheduled for remission-induction chemotherapy for newly diagnosed AML or MDS or in first relapse after remission of at least 6 months, or to start a myeloablative conditioning regimen [26] for a first allogeneic HCT. The main exclusion criteria were clinically documented pneumonia, uncontrolled infection, or previous IFD. Detailed eligibility criteria are provided in the Supplementary File S1.

### Trial Oversight

The trial was sponsored by the EORTC and funded by Merck Sharp & Dohme Corp, a subsidiary of Merck & Co, Inc, Kenilworth, NJ, which also provided caspofungin but had no further role in the trial or in writing the manuscript. The trial statisticians performed the analyses and vouched for the integrity and validity of the analyses. The authors affirm that the trial was conducted as specified in the protocol and agreed with the final manuscript and approved it for publication.

The trial was conducted in accordance with the principles of the Declaration of Helsinki and Good Clinical Practice guidelines and was approved by the institutional review board and ethic committee at each center. All patients provided written informed consent before undergoing any trial-specific procedures. A data review committee (DRC) reviewed eligibility criteria, compliance with the protocol, criteria for IFD, and causes of death. A blinded radiologist reviewed all the CT scans with no or inconclusive reports.

### End Points

The primary end point was overall survival (OS) 42 days after randomization. Key prespecified secondary end points assessed at day 42 and day 84 after randomization included OS, rates of proven or probable IFD (using the 2008 EORTC/National Institute of Allergy and Infectious Diseases Mycoses Study Group (MSG) definitions [27]), compliance with the allocated treatment arm, survival free of proven or probable IFD, number of days of caspofungin administration, and safety. Adverse events (AEs) and serious AEs (SAEs) were assessed according to Common Terminology Criteria for Adverse Events criteria v4.0.

### Randomization and Data Collection

Eligible patients underwent central randomization in a 1:1 ratio using the EORTC online system, stratified according to trial site, allogeneic HCT, and the use of laminar-airflow or high-efficiency particulate-air filtered rooms. Randomization was done within 3 days after the start of chemotherapy or conditioning regimen.

Definitions of major protocol violations are listed in the Supplementary File S2.

### Study Design

#### Arm A (Empiric Arm)

**Figure 1.**
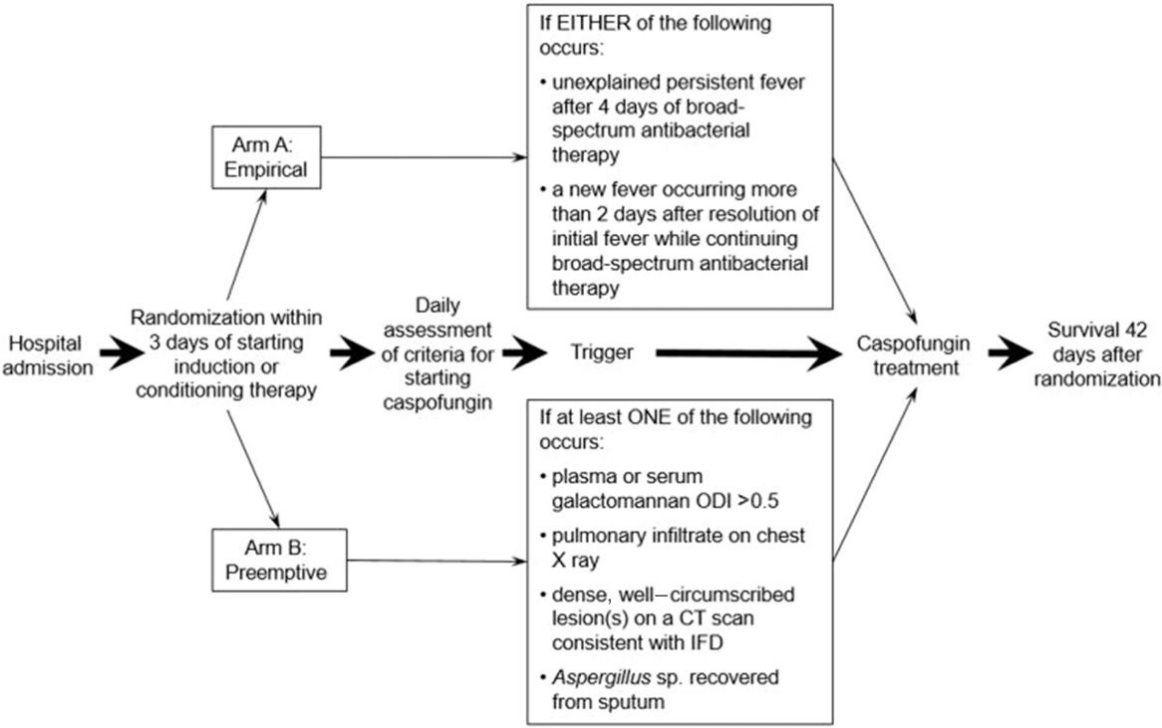
Study design. Abbreviations: CT, computed tomography; IFD, invasive fungal disease; ODI, optical density index

Patients allocated to arm A were given caspofungin either for unexplained fever after 4 days of broad-spectrum antibacterials or for a new febrile episode more than 2 days after resolution of the first episode while continuing broad-spectrum antibacterials (Figure 1). Patients allocated to this study arm were not screened for blood galactomannan before starting caspofungin.

#### Arm B (Preemptive Arm)

Patients in arm B were screened twice weekly on site for blood galactomannan with the enzyme immunoassay (Platelia, Bio-Rad Laboratories, Marnes-La-Coquette, France), according to the manufacturer’s instructions. A positive galactomannan assay (optical density index above 0.5), a new pulmonary infiltrate on chest X ray, or the recovery of *Aspergillus* in sputum prompted a chest CT scan. Patients were given caspofungin when there was a single positive galactomannan test result (even when asymptomatic), a new pulmonary infiltrate on chest X ray and IFD could not be readily excluded per the investigator’s judgment, a new pulmonary infiltrate on chest CT scan consistent with an IFD (a nodule, with or without a halo; a cavity; an air crescent sign), or/and the recovery of *Aspergillus* species by culture from sputum [27].

### Study Procedures

All patients received fluconazole 400 mg/day to prevent *Candida* infection and remained in the hospital for the duration of neutropenia. All patients who developed a first episode of febrile neutropenia (temperature ≥ 38.3°C once or ≥ 38.0°C twice consecutively with an absolute neutrophil count [ANC] <500 neutrophils/mm^3^ or expected to fall within 48 hours) underwent a diagnostic workup with clinical examination, chest X ray, or chest CT scan according to institutional practice; at least 2 separate sets of blood cultures; and appropriate specimens from any other potential sites of infection (as clinically indicated) for microbiology before starting broad-spectrum antibacterials according to local standards and consistent with the Infectious Diseases Society of America guidelines [3].

Once the study arm–specific criteria for starting caspofungin treatment were met, additional blood cultures were taken in both arms, as well as appropriate cultures from other sites, whenever clinically indicated. A chest X ray was ordered for patients in arm A, and a chest CT scan was ordered for patients in arm B. Following the initiation of caspofungin, blood samples for the detection of galactomannan were collected twice weekly in both study arms. Additional examinations, including cultures, imaging, bronchoscopy with bronchoalveolar lavage, and/or needle aspirates or biopsies, were performed on clinical indication.

### Caspofungin

Caspofungin was given at a loading dose of 70 mg on day 1, thereafter 50 mg/day (70 mg if body weight exceeded 80 kg) until neutrophil recovery (ANC ≥ 500 neutrophils/mm^3^) or until the diagnosis of a proven or probable IFD, whichever occurred first. Fluconazole prophylaxis was stopped, and no other systemic antifungal was allowed during administration of caspofungin. In case of proven or probable IFD, caspofungin was stopped and further antifungal therapy was given according to local guidelines.

### Statistical Analyses

Patients were considered eligible if they satisfied all the entry criteria and met none of the exclusion criteria. All eligible patients constituted the modified intention-to-treat (mITT) population and were included in the primary analysis. The per-protocol (PP) population included only cases without any major protocol violation as defined by the DRC.

Our aim in this study was to show that the overall survival 42 days after randomization of the preemptive strategy (arm B) was not inferior to that of the empiric strategy (arm A). Every death was taken into consideration, regardless of the cause, for assessing the primary end point.

We estimated an 87% survival rate at day 42 in arm A based on the results of high-risk patients receiving fluconazole prophylaxis [28]. The greatest relative risk for death acceptable for the noninferiority of arm B was set at 1.62. With a power of 80% for rejecting the null hypothesis, we calculated that 556 patients were needed for the study.

The survival rates at day 42 were estimated in each treatment arm using Kaplan–Meier estimates, and we calculated the survival rate at day 42 of each arm and considered arm B as noninferior to arm A if the ratio of the upper bound 95% confidence interval (CI) for arm B/the lower bound 95% CI of arm A was less than 1.62.

The safety analysis included all randomized patients with the worst degree of toxicity measured between randomization and day 84 being reported. Only the rates of patients developing at least 1 grade 3/4 AE or at least 1 SAE in each randomization arm are reported with 95% CIs.

A planned interim analysis was done by the EORTC Independent Data Monitoring Committee (IDMC) in February 2014 when 263 patients had a follow-up ≥ 42 days. The aim was to determine whether the survival rate in the empiric arm A was in the expected range. The survival rate in the control arm was higher than anticipated (94%; 95% CI, 90%–98%). However, the IDMC recommended that the sample size not be increased as the noninferiority margin that was chosen was stricter than that chosen for the PREVERT trial [23]. In addition, increasing the sample size would have jeopardized recruitment, delayed the trial results, and increased costs.

## RESULTS

**Figure 2.**
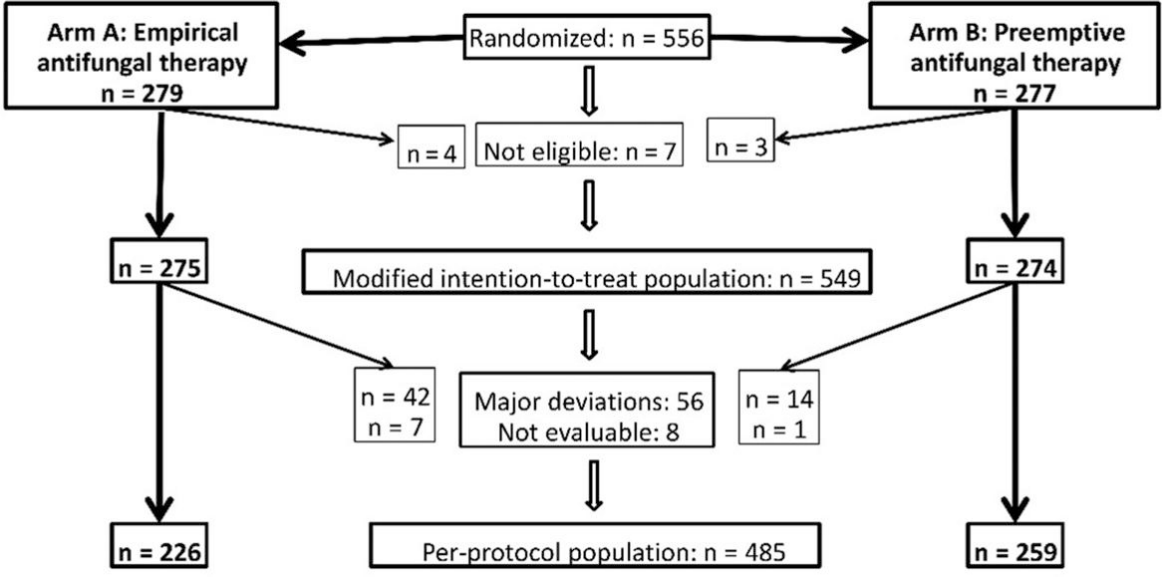
Study results.

From 9 March 2012 to 30 September 2015, 556 patients were randomized at 15 sites in 6 European countries (Supplementary File S3): 279 in arm A and 277 in arm B (Figure 2). Seven patients were found ineligible by the DRC, resulting in an mITT population of 549 patients (Table 1). There was a major protocol deviation (Supplementary File S4) in 56 cases, 42 of 268 (15.7%) in arm A and 14 of 273 (5.1%) in arm B (*P* < .001). Another 8 patients were not evaluable, resulting in a PP population of 485 patients.

**Table 1.** Patient Characteristics of the Modified Intention-to-treat Population

	Arm A: Empirical Antifungal Therapy (n = 275)						Arm B: Preemptive Antifungal Therapy (n = 274)					
					Total						Total	
Characteristic	AML/MDS (n = 222)		Allogeneic HCT (n = 53)		(n = 275)		AML/MDS (n = 216)		Allogeneic HCT (n = 58)		(n = 274)	
Age at randomization, years												
ȃMean ± SD	55.1 ± 13.7		39.4 ± 12.9		52.1 ± 14.8		54.3 ± 13.7		37.7 ± 12.0		50.8 ± 15.0	
ȃMedian (min–max)	59 (18 to 78)		41 (18 to 70)		54 (18 to 78)		58 (18 to 77)		35 (18 to 73)		52.5 (18 to 77)	
Sex												
Male	121	54.5%	32	60.4%	153	55.6%	122	56.5%	36	62.1%	158	57.7%
Female	101	45.5%	21	39.6%	122	44.4%	94	43.5%	22	37.9%	116	42.3%
Underlying disease												
De novo AML	162	73.0%	22	41.5%	184	66.9%	159	73.6%	27	46.6%	186	67.9%
Secondary AML	44	19.8%	4	7.6%	48	17.5%	43	19.9%	4	6.9%	47	17.2%
Myelodysplastic syndrome	14	6.3%	3	5.7%	17	6.2%	14	6.5%	3	5.2%	17	6.2%
Acute lymphoblastic leukemia	…	…	11	20.8%	11	4.0%	…	…	9	15.5%	9	3.3%
Chronic myeloid leukemia	…	…	3	5.7%	3	1.1%	…	…	5	8.6%	5	1.8%
Lymphoma	…	…	4	7.6%	4	1.5%	…	…	3	5.2%	3	1.1%
Multiple myeloma	…	…	1	1.9%	1	0.4%	…	…	4	6.9%	4	1.5%
Aplastic anemia	…	…	…	…	…	…	…	…	1	1.7%	1	0.4%
Other	2	0.9%	5	9.4%	7^a^	2.6%	…	…	2	3.5%	2^a^	0.7%
AML risk classification in AML patients	(n = 206)		(n = 26)		(n = 232)		(n = 202)		(n = 31)		(n = 233)	
Favorable	44	21.8%	1	4.0%	45	19.8%	43	22.1%	2	6.5%	45	19.9%
Intermediate-1	50	24.8%	6	24.0%	56	24.7%	49	25.1%	8	25.8%	57	25.2%
Intermediate-2	35	17.3%	4	16.0%	39	17.2%	42	21.5%	8	25.8%	50	22.1%
Unfavorable	73	36.1%	14	56.0%	87	38.3%	61	31.3%	13	41.9%	74	32.7%
Unknown	4	…	1	…	5	…	7	…	0	…	7	…
AML or MDS treatment phase	(n = 222)	…	…	…	…	(n = 216)	…	…	…	…		
Newly diagnosed, first induction chemotherapy	206	92.8%	…	…	…	…	198	91.7%	…	…	…	…
Relapse	16	7.2%	…	…	…	…	18	8.3%	…	…	…	…
Chemotherapy administered for AML or MDS												
Ara-C (200 mg/m^2^; 7 days) + anthracycline (3 days; idarubicin or daunorubicin)	162	73.0%	…	…	…	…	164	75.9%	…	…	…	…
Intermediate- or high-dose Ara-C	26	11.7%	…	…	…	…	15	6.9%	…	…	…	…
Ara-C + anthracycline + etoposide	17	7.7%	…	…	…	…	14	6.5%	…	…	…	…
Other^b^	17	7.7%	…	…	…	…	23	10.7%	…	…	…	…
Conditioning regimen chemotherapy in allogeneic HCT												
Cyclophosphamide + total body irradiation 12 gray	…	…	29	54.7%	…	…	…	…	30	51.7%	…	…
Busulfan + cyclophosphamide	…	…	14	26.4%	…	…	…	…	13	22.4%	…	…
Etoposide + total body irradiation 10–12 gray	…	…	…	…	…	…	…	…	3	5.2%	…	…
Other^c^	…	…	10	18.9%	…	…	…	…	12	20.7%	…	…
Duration of neutropenia (absolute neutrophil count <0.5 × 10^9^/L), days												
Mean ± SD	24.1 ± 10.8		18.5 ± 5.6		23.1 ± 10.3		23.3 ± 11.5		18.4 ± 6.6		22.2 ± 10.8	
Median (Q1–Q3)	22 (18 to 28)		18 (15 to 22)		22 (18 to 28)		22 (17 to 27)		19 (15 to 22)		21 (17 to 26)	

Abbreviations: AML, acute myeloid leukemia; Ara-C: cytarabine; HCT, hematopoietic cell transplantation; MDS, myelodysplastic syndrome; SD, standard deviation.

Other underlying diseases. Arm A: biphenotypic acute leukemia (n = 1), high-risk myelodysplastic syndrome(n = 1), plasmocytoma (n = 1), primary myelofibrosis (n = 2), myeloid/lymphoid malignancy with eosinophilia (n = 1), sickle-cell disease (n = 1). Arm B: myelodysplastic syndrome with myelofibrosis (n = 1), myelofibrosis complicating polycythemia vera (n = 1).

Other induction chemotherapies for AML or MDS. Arm A: regimens including Ara-C plus idarubicin, daunorubicin, or amsacrine and/or clofarabine or fludarabine (n = 14); regimens including Ara-C, mitoxantrone, and etoposide (n = 1); idarubicin alone (n = 2). Arm B: regimens including Ara-C plus idarubicin, daunorubicin, or amsacrine and/or clofarabine or fludarabine (n = 13); regimens including Ara-C, mitoxantrone, and etoposide (n = 6); idarubicin alone (n= 2); regimen including Ara-C and gemtuzumab ozagamicin (n = 2).

Other conditioning regimens in allogeneic HCT. Arm A: regimens including cyclophosphamide, idarubicin, and total body irradiation (n = 5); regimens including fludarabine and thiotepa (n = 2); regimens including fludarabine and busulfan or cyclophosphamide (n = 3). Arm B: regimens including cyclophosphamide, idarubicin, and total body irradiation (n = 4); regimens including fludarabine and thiotepa (n = 1); regimens including cyclophosphamide and total body irradiation of 9 gray (n = 2); regimens including fludarabine and busulfan or cyclophosphamide (n = 1); other myeloablative regimens (n = 4)

Characteristics of the mITT population (N = 549) are summarized in Table 1. The median duration (Q1–Q3) of neutropenia was 22 days (IQR, 18–28) in arm A and 21 days (IQR 17–26) in arm B (*P* = .15). Characteristics of the PP population (n = 485) are shown in Supplementary Table 1.

### Overall Survival and Causes of Deaths

**Figure 3.**
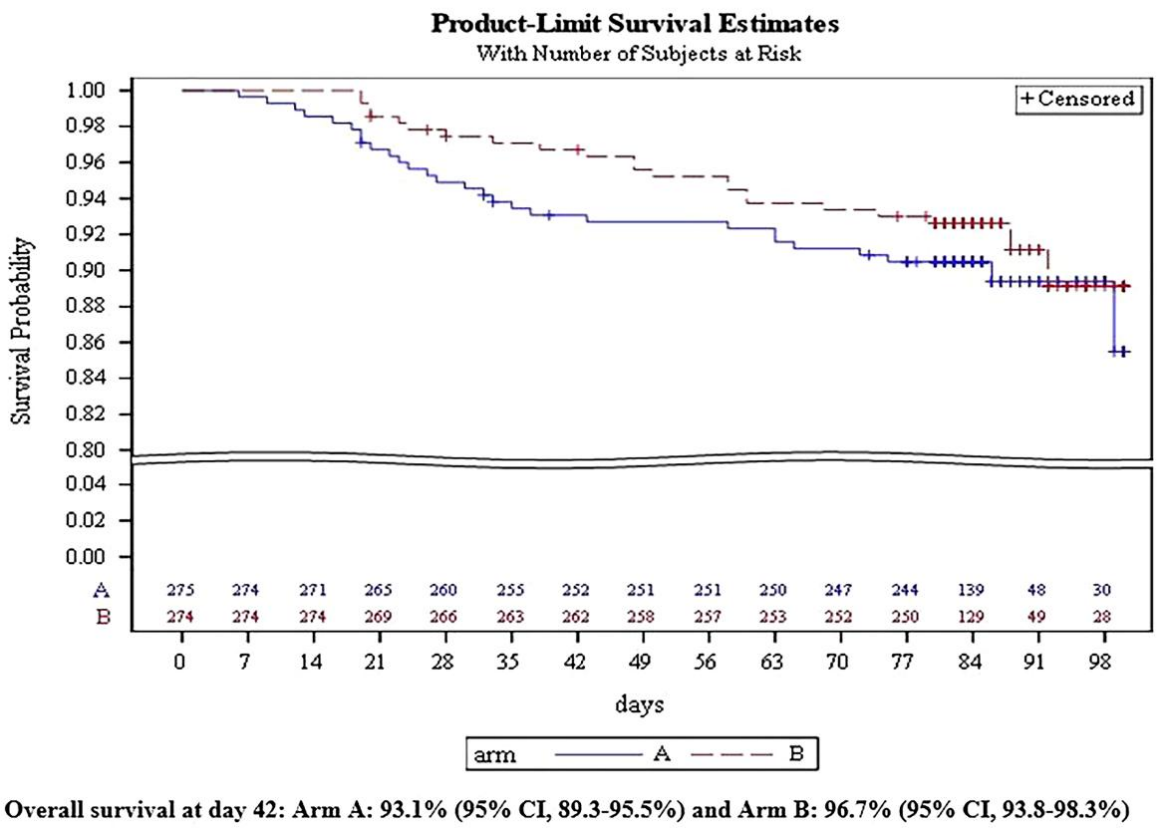
Overall survival in the mITT population (primary endpoint).

The OS in the mITT population at day 42 was not inferior in arm B compared with arm A considering the noninferiority margin of 1.62: arm B (96.7%; 95% CI, 93.8%–98.3%) and arm A (93.1%; 95% CI, 89.3%–95.5%; Figure 3). The OS at day 84 was similar (92.6%; 95% CI, 88.8%–95.2% in arm B vs 90.5%; 95% CI, 86.3%–93.4% in arm A). There was no significant difference in the OS at day 42 in the group of allogeneic HCT recipients (arm B, 94.8%; 95% CI, 84.7%–98.3% vs arm A, 92.4%; 95% CI, 81.0%–97.1%) when compared with those in the AML/MDS patients (arm B, 97.2%; 95% CI, 84.7%–98.3% vs arm ,A 93.2%; 95% CI, 89.0%–95.9%).

In arm B, 20 patients died within 84 days after randomization from IFD plus another cause (n = 5), another cause without IFD (n = 12), or from another cause with an unknown fungal status (n = 3). In arm A, 26 patients died within 84 days after randomization from IFD alone (n = 1), from IFD plus another cause (n = 2), from another cause without IFD (n = 13), and from another cause with an unknown fungal status at time of death (n = 10).

The OS for the PP population in arm B was not inferior to that in arm A (Supplementary Figure 1).

### Invasive Fungal Diseases

The rates of proven and probable IFD within 84 days after randomization were 7.7% in arm B and 6.6% in arm A (*P* = .61; Table 2) in the mITT population. There was no statistically significant difference in the IFD rates between the subgroups of AML/MDS patients and the HCT recipient group. Among the 39 proven or probable IFDs observed, 33 (84.6%) occurred before day 42. Eight of the 11 proven cases and 25 of the 28 probable cases were diagnosed within 42 days after randomization; the remainder were diagnosed between day 42 and day 84. Most proven IFDs were due to *Candida*, and all probable IFDs were aspergillosis.

**Table 2.** Proven and Probable Invasive Fungal Diseases Within 84 Days After Randomization in the Modified Intention-to-Treat Population

Invasive Fungal Disease	Arm A: Empirical Antifungal Therapy			Arm B: Preemptive Antifungal Therapy			*P* Value
	n	Rate	95% CI	n	Rate	95% CI	
Acute myeloid leukemia/myelodysplastic syndrome	(n = 222)			(n = 216)			
Proven	3	1.4%	.3% to 3.9%	6	2.8%	.1% to 6.0%	.33
Probable	13	5.9%	3.2% to 9.8%	12	5.6%	2.9% to 9.5%	1
Proven or probable	16	7.2%	3.8% to 10.6%	18	8.3%	4.7% to 12.0%	.66
Allogeneic hematopoietic cell transplantation	(n = 53)			(n = 58)			
Proven	1	1.9%	.1% to 10.1%	1	1.7%	.0% to 9.2%	1
Probable	1	1.9%	.1% to 10.1%	2	3.5%	.4% to 11.9%	1
Proven or probable	2	3.8%	.5% to 13.0%	3	5.2%	1.1% to 14.4%	1
All	(n = 275)			(n = 274)			
Proven	4	1.5%	.4% to 3.7%	7	2.6%	1.0% to 5.2%	.38
Probable	14	5.1%	2.8% to 8.4%	14	5.1%	2.8% to 8.4%	1
Proven or probable	18	6.6%	3.6% to 9.5%	21	7.7%	4.5% to 10.8%	.61
Causes of proven IFD							
Candidemia	4			5			
*Candida albicans*	1			2			
*Candida nonalbicans*	3			3			
*Geotrichum capitatum*				1			
*Rhizomucor* sp.				1			
Causes of probable IFD							
Aspergillosis	14			14			
Documented by positive culture of BAL	3			1			
*Aspergillus fumigatus*	2			0			
*Aspergillus niger*	1			0			
*Aspergillus* species	0			1			
Documented by cytology in BAL	2			0			
Documented by galactomannan in blood	7			10			
Documented by galactomannan in BAL	2			3			

Abbreviations: BAL, bronchoalveolar lavage; CI, confidence interval; IFD, invasive fungal disease.

### Fungal-Free Survival

In the mITT population, there was no difference in survival free of proven or probable IFD at day 42: arm B, 90.6% (95% CI, 86.3%–93.6%) and arm A, 88.3% (95% CI, 83.8%–91.7%) or at day 84: arm B, 88.6% (95% CI, 83.7%–92.1%) and arm A, 85.5% (95% CI, 80.0%–89.5. Similar features were observed in the PP population (data not shown).

### Antifungal Therapies

The rate at which mITT patients received caspofungin treatment according to the randomized strategy was 27% in arm B vs 63% in arm A (*P* < .001), but with no difference in the caspofungin treatment duration (Table 3). The rates of administration of other antifungals were similar between arms. The number of patients who started preemptive caspofungin on the basis of each triggering criterion (or combination of criteria) is summarized in Supplementary Table 5.

**Table 3.** Antifungal Therapies Administered Within 84 Days After Randomization in the Modified Intention-to-Treat Population

Antifungal Therapy	Arm A (N = 275)	Arm B (N = 274)	*P* Value
Caspofungin administered according to the study protocol			
No. of treated patients (%)	173 (63)	73 (27)	<.001
Median duration of treatment (IQR, Q1–Q3), wks	1.7 (1.0–2.7)	1.4 (0.7–2.4)	.36
Caspofungin or other echinocandins administered outside of the study protocol			
No. of treated patients (%)	10 (3.6)	11 (4.0)	.83
Mold active azole			
No. of treated patients (%)	86 (31.3)	75 (27.4)	.32
Median duration of treatment (IQR, Q1–Q3), wks	4.2 (1.6–8.1)	3.0 (1.4–9.6)	.67
Intravenous amphotericin B			
No. of treated patients (%)	26 (9.5)	28 (10.2)	.76
Median duration of treatment (IQR, Q1–Q3), wks	1.9 (1.3–3.0)	1.1 (0.9–2.4)	.07

Abbreviation: IQR, interquartile range.

### Safety

The rates for patients experiencing at least 1 grade 3, 4, or 5 AE or at least 1 SAE were not different between arms (Supplementary File S6).

## DISCUSSION

This study shows that a preemptive antifungal strategy that includes twice weekly galactomannan screening and CT scan on demand does not prejudice the overall survival of adults with prolonged neutropenia who are at high risk for IFD while receiving fluconazole prophylaxis. In addition, is the strategy is not associated with an increased risk of proven or probable IFD. Indeed, the strategy reduces the use of antifungals by half, which should prove cost-saving.

This study adds important information to previous trials, especially the French PREVERT study that was not sufficiently powered to establish the noninferiority of the preemptive strategy in the subgroup of patients receiving AML induction chemotherapy [23].

Several differences should be noted between the 2 trials. The present study was exclusively focused on long-term neutropenia; allogeneic HCT recipients were included; all patients received fluconazole prophylaxis; the IFDs were defined according to the EORTC/MSG 2008 consensus definitions [27], not the earlier version [29]; and caspofungin was used exclusively for both empiric and preemptive therapy. In the PREVERT study, the rate of IFDs in the AML-induction group (with a median duration of neutropenia of 26 days) that was preemptively managed was 16.4% and significantly higher than the rate of the empiric group (3.8%). Although this difference could partly be explained by more diagnostic procedures used in the preemptive arm, it could also be due to use of the original 2002 EORTC/MSG definitions rather than the revised 2008 definitions. It could also be attributed to an increased risk for IFD due to the administration of antifungals to fewer patients for a shorter time in the preemptive strategy.

Others also reported an excess risk for IFD with a preemptive strategy [16, 21]. Even though an AML patient may initially survive the IFD, having an IFD impacts the long-term outcome due to the risk of IFD recurrence, leading to modifying or postponing subsequent courses [30]. Hence, relying only on survival of less than 3 months could miss the consequences of IFD on the final outcome. In the present study, we did not observe any excess IFDs with the experimental strategy, so this fear can be allayed. With an IFD rate of 7% in the AML group, our results are consistent with the 8% rate in the Prospective Invase Mould Disease Audit (PIMDA) study [31]. Similarly, our IFD rate of 4.5% assessed within 12 weeks after starting the conditioning regimen in HCT recipients is consistent with recent data showing that currently two-thirds of the aspergillosis cases observed after allogeneic HCT occur after day 100 post-transplant [32].

The preemptive strategy is applicable as long as the center uses routine screening of galactomannan, has ready access to CT scans, and the costs are balanced by the reduced use of antifungals. Today, many centers use some hybrid strategy, mixing empiric administration of antifungals and a biomarker or imaging screening, which adds the cost of overuse of antifungals to the costs of biologic screening and CT scan.

Our study has several strengths. First, we chose survival as a hard and objective primary end point, considering that survival is a prerequisite for a favorable outcome. This end point highlights the need for the best strategies to be used during high-risk neutropenia. Second, our groups were well balanced in terms of AML risk and neutropenia duration, precluding any impact of these parameters on survival. Third, we assessed only proven and probable IFDs and did not consider possible IFDs where fungal causality is less certain. Last, the size of the AML cohort allowed us to be confident of the overall results and to generalize them to such patients as a whole.

No study is free of limitations. First, the comparison of 2 strategies with different diagnostic workup and triggering factors for antifungal administration made a blinded design impossible to apply. Second, we chose not to use antimold prophylaxis because it lowers the sensitivity of galactomannan for screening. So, while our preemptive strategy would not be suitable for those centers that do use antimold prophylaxis [33], either strategy would be useful to those centers that do not use it or in patients who cannot continue on mold-active azole prophylaxis due to AEs or clinically important drug–drug interactions.

Empiric antifungal therapy has been the gold standard for managing IFD in neutropenic patients and will remain so for centers with limited diagnostic resources that rely on a clinically driven approach. The results of our study now provide a viable alternative.

## Supplementary Data

Supplementary materials are available at *Clinical Infectious Diseases* online. Consisting of data provided by the authors to benefit the reader, the posted materials are not copyedited and are the sole responsibility of the authors, so questions or comments should be addressed to the corresponding author.

## Supplementary Material

ciac623_Supplementary_Data

## Notes

***Acknowledgments.*** The authors thank the patients and their families and the healthcare providers who participated in the trial.

***Financial support.*** This work was supported by Merck Sharp & Dohme Corp, USA, through an educational grant.

## References

[1] EORTC International Antimicrobial Therapy Cooperative Group . Empiric antifungal therapy in febrile granulocytopenic patients. Am J Med 1989; 86:668–72. 2658574 10.1016/0002-9343(89)90441-5

[2] Pizzo P , Robichaud KJ, Gill FA, Witebsky FG. Empirical antibiotic and antifungal therapy for cancer patients with prolonged fever and granulocytopenia. Am J Med 1982; 72:101–11. 7058815 10.1016/0002-9343(82)90594-0

[3] Freifeld AG , Bow EJ, Sepkowitz KA, et al Clinical practice guidelines for the use of antimicrobial agents in neutropenic patients with cancer: 2010 update by the Infectious Diseases Society of America. Clin Infect Dis 2011; 52:427–31. 21205990 10.1093/cid/ciq147

[4] Marchetti O , Cordonnier C, Calandra T. Empirical antifungal therapy in neutropaenic cancer patients with persistent fever. Eur J Cancer 2007; 5:32–42.

[5] Walsh TJ , Finberg RW, Arndt C, et al Liposomal amphotericin B for empirical therapy in patients with persistent fever and neutropenia. N Engl J Med 1999; 340:764–71. 10072411 10.1056/NEJM199903113401004

[6] Walsh TJ , Teppler H, Donowitz GR, et al Caspofungin vs liposomal amphotericin B for empirical antifungal therapy in patients with persistent fever and neutropenia. N Engl J Med 2004; 351:1391–402. 15459300 10.1056/NEJMoa040446

[7] Bretagne S , Marmorat-Khuong A, Kuentz M, Latge JP, Bart-Delabesse E, Cordonnier C. Serum *Aspergillus galactomannan* antigen testing by sandwich ELISA: practical use in neutropenic patients. J Infect 1997; 35:7–15. 9279718 10.1016/s0163-4453(97)90833-1

[8] Maertens J , Van Eldere J, Verhaegen J, Verbeken E, Verschakelen J, Boogaerts M. Use of circulating galactomannan screening for early diagnosis of invasive aspergillosis in allogeneic stem cell transplant recipients. J Infect Dis 2002; 186:1297–306. 12402199 10.1086/343804

[9] Maertens J , Verhaegen J, Demuynck H, et al Autopsy-controlled prospective evaluation of serial screening for circulating galactomannan by a sandwich enzyme-linked immunosorbent assay for haematological patients at risk for invasive aspergillosis. J Clin Microbiol 1999; 37:3223–8. 10488181 10.1128/jcm.37.10.3223-3228.1999 PMC85532

[10] Mennink-Kersten MA , Donnelly JP, Verweij PE. Detection of circulating galactomannan for the diagnosis and management of invasive aspergillosis. Lancet Infect Dis 2004; 4:349–57. 15172343 10.1016/S1473-3099(04)01045-X

[11] Caillot D , Couaillier JF, Bernard A, et al Increasing volume and changing characteristics of invasive pulmonary aspergillosis on sequential thoracic computed tomography scans in patients with neutropenia. J Clin Oncol 2001; 19:253–9. 11134220 10.1200/JCO.2001.19.1.253

[12] Heussel CP , Kauczor HU, Heussel GE, et al Pneumonia in febrile neutropenic patients and in bone marrow and blood stem-cell transplant recipients: use of high-resolution computed tomography. J Clin Oncol 1999; 17:796–805. 10071269 10.1200/JCO.1999.17.3.796

[13] Maertens J , Theunissen K, Verhoef G, et al Galactomannan and computed tomography-based preemptive antifungal therapy in neutropenic patients at high risk for invasive fungal infection: a prospective feasibility study. Clin Infect Dis 2005; 41:1242–50. 16206097 10.1086/496927

[14] Aguilar-Guisado M , Espigado I, Cordero E, et al Empirical antifungal therapy in selected patients with persistent febrile neutropenia. Bone Marrow Transplant 2010; 45:159–64. 19525983 10.1038/bmt.2009.125

[15] Dignan FL , Evans SO, Ethell ME, et al An early CT-diagnosis based treatment strategy for invasive fungal infection in allogeneic transplant recipients using caspofungin first line: an effective strategy with low mortality. Bone Marrow Transplant 2009; 44:51–6. 19139735 10.1038/bmt.2008.427

[16] Pagano L , Caira M, Nosari A, et al The use and efficacy of empirical vs preemptive therapy in the management of fungal infections: the HEMA e-Chart Project. Haematologica 2011; 96:1366–70. 21565903 10.3324/haematol.2011.042598 PMC3166108

[17] Tan B , Low J, Chlebicka N, et al Galactomannan-guided preemptive vs. empirical antifungals in the persistently febrile neutropenic patient: a prospective randomized study. Int J Infect Dis 2011; 15:e350–6. 21397541 10.1016/j.ijid.2011.01.011

[18] Blenow O , Remberger M, Klingspor L, et al Randomized PCR-based therapy and risk factors for invasive fungal infection following reduced-intensity conditioning and hematopoietic SCT. Bone Marrow Transplant 2010; 45:1710–8. 20190840 10.1038/bmt.2010.38

[19] Cuenca-Estrella M , Meije Y, Diaz-Pedroche C, et al Value of serial quantification of fungal DNA by a real-time PCR based technique for early diagnosis of invasive aspergillosis in patients with febrile neutropenia. J Clin Microbiol 2009; 47:379–84. 19109479 10.1128/JCM.01716-08 PMC2643681

[20] Halliday C , Hoile R, Sorrell T, et al Role of prospective screening of blood for invasive aspergillosis by polymerase chain reaction in febrile neutropenic recipients of haematopoietic stem cell transplants and patients with acute leukaemia. Br J Haematol 2005; 132:478–86. 10.1111/j.1365-2141.2005.05887.x 16412020

[21] Morissey O , Chen S, Sorrell T, et al Galactomannan and PCR vs culture and histology for directing use of antifungal treatment for invasive aspergillosis in high-risk hematology patients: a randomised controlled trial. Lancet Infect Dis 2013; 13:519–28. 23639612 10.1016/S1473-3099(13)70076-8

[22] Girmenia C , Micozzi A, Gentile G, et al Clinically driven diagnostic antifungal approach in neutropenic patients: a prospective feasibility study. J Clin Oncol 2010; 28:667–74. 19841328 10.1200/JCO.2009.21.8032

[23] Cordonnier C , Pautas C, Maury S, et al Empirical vs preemptive antifungal therapy for high-risk, febrile, neutropenic patients: a randomized, controlled trial. Clin Infect Dis 2009; 48:1042–51. 19281327 10.1086/597395

[24] Kimura SI , Murata T, Akahoshi Y, et al Economic evaluation of a preemptive treatment strategy for invasive fungal infection in neutropenic patients with hematological diseases. Eur J Clin Microbiol Infect Dis 2015; 34:951–61. 25577175 10.1007/s10096-014-2311-8

[25] Severens J , Donnelly J, Meris J, De Pauw B, Verweij P. Two strategies for managing invasive aspergillosis: a decision analysis. Clin Infect Dis 1997; 25:1148–54. 9402374 10.1086/516085

[26] Transplantation ESfBaM. MED-AB FORMS MANUAL: A guide to the completion of the EBMT HSCT Med-AB forms. The European Society for Blood and Marrow Transplantation.

[27] De Pauw B , Walsh T, Donnelly JP, et al Revised definitions of invasive fungal disease from the European Organization for Research and Treatment of Cancer/Invasive Fungal Infections Cooperative Group and the National Institute of Allergy and Infectious Diseases Mycoses Study Group (EORTC/MSG) Consensus Group. Clin Infect Dis 2008; 46:1813–21. 18462102 10.1086/588660 PMC2671227

[28] Cornely OA , Maertens J, Winston DJ, et al Posaconazole vs. fluconazole or itraconazole prophylaxis in patients with neutropenia. N Engl J Med 2007; 356:348–59. 17251531 10.1056/NEJMoa061094

[29] Ascioglu S , Rex JH, de Pauw B, et al Defining opportunistic invasive fungal infections in immunocompromised patients with cancer and hematopoietic stem cell transplants: an international consensus. Clin Infect Dis 2002; 34:7–14. 11731939 10.1086/323335

[30] Even C , Bastuji-Garin S, Hicheri Y, et al Impact of invasive fungal disease on the chemotherapy schedule and event-free survival in acute leukemia patients who survived fungal disease: a case-control study. Haematologica 2011; 96:337–41. 21071502 10.3324/haematol.2010.030825 PMC3031706

[31] Donnelly J , Cordonnier C, Cuenca-Estrella M, et al A European period prevalence study to estimate the rate of invasive pulmonary mould disease (PIMDA Study). Barcelona, Spain: ECCMID, 2014.

[32] Robin C , Cordonnier C, Sitbon K, et al Mainly post-transplant factors are associated with invasive aspergillosis after allogeneic stem cell transplantation: a study from the Surveillance des Aspergilloses Invasives en France and Société Francophone de Greffe de Moelle et de Thérapie Cellulaire. Biol Blood Marrow Transplant 2019; 25:354–61. 30268782 10.1016/j.bbmt.2018.09.028

[33] Duarte RF , Sánchez-Ortega I, Cuesta I, et al Serum galactomannan-based early detection of invasive aspergillosis in hematology patients receiving effective antimold prophylaxis. Clin Infect Dis 2014; 59:1696–702. 25165088 10.1093/cid/ciu673

