# Supplementary material for: Empiric vs Preemptive Antifungal Strategy in High-Risk Neutropenic Patients on Fluconazole Prophylaxis: A Randomized Trial of the European Organization for Research and Treatment of Cancer
Source: Clin Infect Dis. 2022 Jul 30;76(4):674–82. doi: 10.1093/cid/ciac623 (PMC9938744; doi:10.1093/cid/ciac623)
Supplement: ciac623_Supplementary_Data [file ciac623_supplementary_data.docx]

**Empiric versus pre-emptive antifungal strategy in high-risk neutropenic patients on fluconazole prophylaxis: a randomized trial of the European organization for Research and Treatment of cancer (EORTC 65091)**

**Supplementary Appendix**

**Table of contents**

S1 Detailed eligibility criteria

S2 Protocol violations-definitions

S3 List of participating centers and number of subjects included

S4 Major protocol violations

S5 Triggers for starting caspofungin in arm B (pre-emptive)

S6 Adverse events

Table S1 Baseline characteristics of the per-protocol population

Figure S1 Overall survival in the per-protocol population

Figure S2 Timing of the proven/probable IFD by arm.

S1 Detailed eligibility criteria

Selection criteria have to be checked within 3 days before randomization once patients are admitted to the hospital. The patient must fulfil all of the following criteria:

♦ Age 18 years or older.

♦ Start within 3 days before randomization, remission induction chemotherapy for acute myeloid leukemia (AML) or myelodysplastic syndrome (MDS) that is newly diagnosed or in first relapse after hematological remission lasting for a minimum duration of 6 months OR start within 3 days before randomization, myeloablative conditioning regimen to prepare for a first allogeneic HSCT. Permissible conditioning regimens are outlined in Appendix E.

♦ Planned hospital admission for the duration of the neutropenic phase (ANC< 0.5 x 109 /L). ♦ Planned oral or intravenous (I.V.) fluconazole for Candida prophylaxis at the dose of 400 mg/day; no other antifungal systemic prophylaxis is allowed.

♦ No previous or current history of proven or probable IFD.

♦ No current clinical diagnosis of pneumonia.

♦ No serious uncontrolled concomitant disease or comorbidity that, in the opinion of the investigator, may compromise adherence to the study protocol.

♦ No history of allergy or any adverse reaction to echinocandin drugs (caspofungin, micafungin, or anidulafungin).

♦ No inadequately treated infection at study entry.

♦ No documented HIV infection.

♦ No concomitant inclusion on other clinical trial using an investigational drug for infectious diseases.

♦ Female subjects with childbearing potential must have a negative serum (or urine) pregnancy test within 3 days before randomization.

♦ Female and male subjects of childbearing / reproductive potential should use adequate birth control measures during the treatment period and for at least 3 months after the last study treatment. Adequate birth control measures should be understood highly effective methods as defined by the Note 3 of the note for guidance on non-clinical safety studies for the conduct of human clinical trials for pharmaceuticals (CPMP/ICH/286/95):"A highly effective method of birth control is defined as those which result in low failure rate (i.e. less than 1% per year) when used consistently and correctly such as implants, injectables, combined oral contraceptives, sexual abstinence or vasectomised partner. For subjects using a hormonal contraceptive method, information regarding the product under evaluation and its potential effect on contraceptives should be addressed."

♦ Female subjects who are breast feeding should discontinue nursing prior to randomization. ♦ Absence of any psychological, familial, sociological or geographical condition potentially hampering compliance with the study protocol and follow-up schedule; those conditions should be discussed with the patient before registration in the trial.

♦ Before patient registration/randomization, written informed consent must be given according to ICH/GCP, and national/local regulations.

S2 Protocol violations-definitions

Protocol violations are defined at least by one of these criteria:

- For both arms: addition of any antifungal to the study drug before 14 days of study treatment has elapsed.
- For empirical antifungal therapy (Arm A):
- administration of caspofungin or any other systemic antifungal before unexplained persistent febrile neutropenia has been documented without there being any evidence of IFD.
- failure to start caspofungin despite unexplained persistent febrile neutropenia, or recurrent fever.
- patient who is started on caspofungin when there is neither persistent, nor recurrent fever, nor criteria for proven or probable IFD.
- For pre-emptive arm (Arm B):
- administration of caspofungin without any of the criteria being met for starting study treatment according the study protocol.
- failure to start caspofungin despite criteria for starting study therapy are met.
- failure to screen for galactomannan during the study period.

S3 List of participating centers and number of subjects included

| **Name** | **Affiliation** | **Subjects included** |
| --- | --- | --- |
| Johan A Maertens | University Hospitals, Leuven, BE | 95 |
| Sylvain Chantepie | Hôpital Côte de Nacre, Caen, FR | 70 |
| Christine Robin | Henri Mondor, Créteil, FR | 61 |
| Nicole Blijlevens | Radboud UMC, Nijmegen, NL | 52 |
| Pascal Turlure | Centre Hospitalier Universitaire Limoges, Limoges, FR | 43 |
| Dominik Selleslag | AZ St Jan, Brugge, BE | 40 |
| Frédéric Baron | University of Liège and University Hospital of Liège, Liège, BE | 36 |
| Mickael Aoun | Institut Jules Bordet, Brussels, BE | 33 |
| Werner J Heinz | Universitätsklinikum, Würzburg, DE | 28 |
| Harmut Bertz | Universitätsklinikum, Freiburg, DE | 25 |
| Zdenik Racil | Masaryk University Brno and Institute of Hematology and Blood Transfusion, Prague, CZ | 19 |
| Bernard Vandercam | Cliniques Universitaires St. Luc, Brussels, BE | 17 |
| Lubos Drgona | National Cancer Institute, Bratislava, SK | 16 |
| Valérie Coiteux | Centre Hospitalier Régional Universitaire Lille, Lille, FR | 13 |
| Cristina Castilla Llorente | Institut Gustave Roussy, Villejuif, FR | 8 |

S4 Major protocol violations

| **Arm** |  | **classification** | **Number of cases** |
| --- | --- | --- | --- |
| A | Myeloablative conditioning regimen | Study drug not given | 4 |
| A | Myeloablative conditioning regimen | Study drug started late | 1 |
| A | Myeloablative conditioning regimen | Wrong treatment | 1 |
|  |  |  |  |
| A | Remission induction chemotherapy | Study drug not given | 11 |
| A | Remission induction chemotherapy | Study drug started late | 14 |
| A | Remission induction chemotherapy | Study drug started wrongly | 2 |
| A | Remission induction chemotherapy | Wrong treatment | 9 |
|  |  |  |  |
| B | Myeloablative conditioning regimen | Inadequate screening | 1 |
| B | Myeloablative conditioning regimen | Study drug not given | 1 |
| B | Myeloablative conditioning regimen | Study drug started late | 1 |
| B | Myeloablative conditioning regimen | Wrong treatment | 3 |
|  |  |  |  |
| B | Remission induction chemotherapy | Inadequate screening | 3 |
| B | Remission induction chemotherapy | Study drug not given | 2 |
| B | Remission induction chemotherapy | Study drug started late | 2 |
| B | Remission induction chemotherapy | Study drug started wrongly | 1 |

S5: Triggers for starting caspofungin in arm B (pre-emptive)

| Plasma or serum galactomannan ODI > 0.5 | *Aspergillus* sp. recovered from sputum | dense well circumscribed lesion(s) on a CT scan consistent with IFD | pulmonary infiltrate on chest-X ray | N |
| --- | --- | --- | --- | --- |
| + | + | + | + | 1 |
| + |  | + | + | 12 |
| + |  | + |  | 1 |
| + |  |  | + | 3 |
| + |  |  |  | 9 |
|  | + |  | + | 1 |
|  | + |  |  | 1 |
|  |  | + | + | 24 |
|  |  | + |  | 6 |
|  |  |  | + | 15 |

TOTAL 73

S6 : Adverse events

|  | Arm A  (N = 279) | | | | Arm B  (N = 277) | | | |
| --- | --- | --- | --- | --- | --- | --- | --- | --- |
|  |  | |  | |  | |  | |
|  | **N** | **%** | **95% CI** | | **N** | **%** | **95% CI** | |
| **Patients with at least one grade 3, 4, or 5 AE** | 158 | 57% | 51% to 62% | | 154 | 56% | 50% to 61% | |
|  |  |  |  |  |  |  |  |  |
| **Worst grade* of AEs** observed in at least 2% of patients in either arm A or B, and sort by descending order in arm A | **Grade ≥3** | | **Grade ≥1** | | **Grade ≥3** | | **Grade ≥1** | |
|  | **N** | **%** | **N** | **%** | **N** | **%** | **N** | **%** |
|  | **158** | **57%** | **277** | **99%** | **154** | **56%** | **271** | **98%** |
|  |  |  |  |  |  |  |  |  |
| Infections and infestations | 75 | 27% | 138 | 49% | 64 | 23% | 130 | 47% |
| Gastrointestinal disorders | 58 | 21% | 203 | 73% | 50 | 18% | 206 | 74% |
| Respiratory, thoracic and mediastinal disorders | 31 | 11% | 141 | 51% | 23 | 8% | 133 | 48% |
| General disorders and administration site conditions | 28 | 10% | 156 | 56% | 22 | 8% | 145 | 52% |
| Metabolism and nutrition disorders | 26 | 9% | 50 | 18% | 18 | 6% | 40 | 14% |
| Vascular disorders | 22 | 8% | 82 | 29% | 23 | 8% | 75 | 27% |
| Blood and lymphatic system disorders | 20 | 7% | 35 | 13% | 27 | 10% | 35 | 13% |
| Nervous system disorders | 14 | 5% | 95 | 34% | 19 | 7% | 90 | 32% |
| Investigations | 14 | 5% | 33 | 12% | 10 | 4% | 26 | 9% |
| Musculoskeletal and connective tissue disorders | 14 | 5% | 91 | 33% | 6 | 2% | 61 | 22% |
| Cardiac disorders | 9 | 3% | 43 | 15% | 9 | 3% | 41 | 15% |
| Psychiatric disorders | 6 | 2% | 68 | 24% | 6 | 2% | 72 | 26% |
| Skin and subcutaneous tissue disorders | 4 | 1% | 132 | 47% | 8 | 3% | 149 | 54% |
| Hepatobiliary disorders | 4 | 1% | 12 | 4% | 8 | 3% | 18 | 6% |
| Renal and urinary disorders | 3 | 1% | 47 | 17% | 8 | 3% | 37 | 13% |
| Immune system disorders | 4 | 1% | 27 | 10% | 7 | 3% | 32 | 12% |
|  | **N** | **%** | **95% CI** | | **N** | **%** | **95% CI** | |
| **Patients with at least one SAE** | 62 | 22% | 17% to 27% | | 58 | 21% | 16% to 26% | |

Adverse events of grade > 3 according to the Common Terminology Criteria for Adverse Events v4.0. The safety analyses included all randomized patients (n=556), whether they received caspofungin or not, and collected all AEs from randomization till day 84.

**Table S1** : Baseline characteristics of the per-protocol population

|  | **Arm A: Empirical antifungal therapy**  **(N = 226)** | | | | | | | **Arm B: Pre-emptive antifungal therapy**  **(N = 259)** | | | | | | |
| --- | --- | --- | --- | --- | --- | --- | --- | --- | --- | --- | --- | --- | --- | --- |
|  |  | | | | **Total** | |  | | | | | **Total** | |  |
|  | **AML/MDS**  **(N = 179)** | | **Allogeneic**  **HCT**  **(N = 47)** | | **(N = 226)** | | **AML/MDS**  **(N = 207)** | | | **Allogeneic HCT**  **(N = 52)** | | **(N = 259)** | |  |
|  |  |  |  |  |  |  |  | |  |  |  |  |  |  |
| **Age at randomization** |  | |  | |  | |  | | |  | |  | |  |
| Mean ± standard deviation | 55.7 ± 13.5 | | 39.6 ± 13.1 | | 52.3 ± 14.9 | | 54.1 ± 13.7 | | | 38.1 ± 12.3 | | 50.8 ± 14.9 | |  |
| Median (min-max) | 60 (18 to 78) | | 41 (18 to 70) | | 55 (18 to 78) | | 57 (18 to 77) | | | 36 (18 to 73) | | 53 (18 to 77) | |  |
| **Sex** |  |  |  |  |  |  |  | |  |  |  |  |  |  |
| Male | 103 | 57.5% | 27 | 57.5% | 130 | 57.5% | 116 | | 56.0% | 31 | 59.6% | 147 | 56.8% |  |
| Female | 76 | 42.5% | 20 | 42.6% | 96 | 42.5% | 91 | | 44.0% | 21 | 40.4% | 112 | 43.2% |  |
|  |  |  |  |  |  |  |  | |  |  |  |  |  |  |
| **Underlying disease** |  |  |  |  |  |  |  | |  |  |  |  |  |  |
| De novo AML | 130 | 72.6% | 20 | 42.6% | 150 | 66.4% | 154 | | 74.4% | 26 | 50.0% | 180 | 69.5% |  |
| Secondary AML | 36 | 20.1% | 4 | 8.5% | 40 | 17.7% | 39 | | 18.8% | 3 | 5.8% | 42 | 16.2% |  |
| Myelodysplastic syndrome | 11 | 6.1% | 3 | 6.4% | 14 | 6.2% | 14 | | 6.8% | 3 | 5.8% | 17 | 6.6% |  |
| ALL | - | - | 11 | 20.8% | 11 | 4.0% | - | | - | 9 | 15.5% | 9 | 3.3% |  |
| CML | - | - | 3 | 5.7% | 3 | 1.1% | - | | - | 5 | 8.6% | 5 | 1.8% |  |
| Lymphoma | - | - | 4 | 7.6% | 4 | 1.5% | - | | - | 3 | 5.2% | 3 | 1.1% |  |
| Multiple myeloma | - | - | 1 | 1.9% | 1 | 0.4% | - | | - | 4 | 6.9% | 4 | 1.5% |  |
| Aplastic anemia | - | - | - | - | - | - | - | | - | 1 | 1.7% | 1 | 0.4% |  |
| Other* | 2 | 0.9% | 5 | 9.4% | 7 | 2.6% | - | | - | 2 | 3.5% | 2 | 0.7% |  |

|  |  |  |  |  |  |  |  |  |  |  |  |  |
| --- | --- | --- | --- | --- | --- | --- | --- | --- | --- | --- | --- | --- |
| **AML risk classification in AML patients** † | (N=166) | | (N=24) | | (N = 190) | | (N=193) | | (N=29) | | (N = 222) | |
| Favourable risk | 35 | 21.5% | 1 | 4.4% | 36 | 19.4% | 35 | 21.5% | 1 | 4.4% | 36 | 19.4% |
| Intermediate-1 risk | 44 | 27.0% | 6 | 26.1% | 50 | 26.9% | 44 | 27.0% | 6 | 26.1% | 50 | 26.9% |
| Intermediate-2 risk | 29 | 17.8% | 3 | 13.0% | 32 | 17.2% | 29 | 17.8% | 3 | 13.0% | 32 | 17.2% |
| Unfavourable risk | 55 | 33.7% | 13 | 56.5% | 68 | 36.6% | 55 | 33.7% | 13 | 56.5% | 68 | 36.6% |
| Unknown | 3 |  | 1 |  | 4 |  | 3 |  | 1 |  | 4 |  |
|  |  |  |  |  |  |  |  |  |  |  |  |  |
| **AML or myelodysplastic syndrome treatment phase** | (N=179) | |  |  |  |  | (N=207) | |  |  |  |  |
| Newly diagnosed, first induction | 167 | 93.3% |  |  |  |  | 189 | 91.3% |  |  |  |  |
| Relapse | 12 | 6.7% |  |  |  |  | 18 | 8.7% |  |  |  |  |
| **Chemotherapy administered for AML or myelodysplastic syndrome** |  |  |  |  |  |  |  |  |  |  |  |  |
| Ara-C (200mg/m2) (7d) + anthracyclin (3d) (idarubicin or daunorubicin) | 131 | 73.2% |  |  |  |  | 158 | 76.3% |  |  |  |  |
| Intermediate or high dose Ara-C | 18 | 10.1% |  |  |  |  | 15 | 7.2% |  |  |  |  |
| Ara-C + anthracyclin + etoposide | 14 | 7.8% |  |  |  |  | 14 | 6.8% |  |  |  |  |
| Other‡ | 16 | 8.9% |  |  |  |  | 20 | 9.7% |  |  |  |  |
| **Conditioning regimen chemotherapy in allogeneic HCT** |  |  |  |  |  |  |  |  |  |  |  |  |
| Busulfan + cyclophosphamide |  |  | 13 | 27.7% |  |  |  |  | 12 | 23.1% |  |  |
| Cyclophosphamide + total body irradiation 12 Gray |  |  | 26 | 55.3% |  |  |  |  | 27 | 51.9% |  |  |
| Etoposide + total body irradiation 10-12 Gray |  |  | - | - |  |  |  |  | 2 | 3.9% |  |  |
| Other § |  |  | 8 | 17.0% |  |  |  |  | 11 | 21.2% |  |  |
|  |  |  |  |  |  |  |  |  |  |  |  |  |
|  |  |  |  |  |  |  |  |  |  |  |  |  |
| **Duration of neutropenia (ANC < 0.5 x10^9^/L)(number of days)** |  |  |  |  |  |  |  |  |  |  |  |  |
| Mean ± standard deviation | 23.4±10.4 | | 18.6±5.8 | | 22.4 ±9.8 | | 23.2±11.5 | | 18.7±6.7 | | 22.3±10.8 | |
| Median (Q1-Q3) | 22 (18 to 28) | | 18 (15 to 22) | | 22 (18 to 27) | | 22 (17 to 27) | | 19 (15 to 22) | | 22 (17 to 26) | |

Abbreviations: AML: acute myeloid leukemia. ALL: acute lymphoblastic leukemia. CML: Chronic myeloid leukemia. HCT: Hematopoietic cell transplantation. Ara-C: cytarabine.

*Other underlying diseases: Arm A: biphenotypic acute leukemia (n=1); high-risk myelodysplastic syndrome(n=1); plasmocytoma (n=1); primary myelofibrosis (n=2);), sickle-cell disease (n=1). Arm B: myelodysplastic syndrome with myelofibrosis (n=1), myelofibrosis complicating polycythemia vera (n=1).

‡ Other induction chemotherapies for AML or MDS: Arm A: regimens including Ara-C plus idarubicin, daunorubicin or amsacrine and/or clofarabine or fludarabine (n=13); regimens including Ara-C, mitoxantrone and etoposide (n=1); idarubicin alone (n=2). Arm B: regimens including Ara-C plus idarubicin, daunorubicin or amsacrine and/or clofarabine or fludarabine (n=13); regimens including Ara-C, mitoxantrone and etoposide (n=3); idarubicin alone (n= 2); regimen including Ara-C and gemtuzumab ozagamicin (n=2).

§ Other conditioning regimens in allogeneic HCT: Arm A: regimens including cyclophosphamide, idarubicin, and total body irradiation(n=4); regimens including fludarabine and thiotepa (n=1); regimens including fludarabine and busulfan or cyclophosphamide (n=3). Arm B: regimens including cyclophosphamide, idarubicin, and total body irradiation (n=4); regimens including fludarabine and thiotepa (n=1); regimens including cyclophosphamide and total body irradiation of 9 Gray (n=2) ; regimens including fludarabine and busulfan or cyclophosphamide (n=3); other myeloablative regimen (n=1).

Figure S1 Overall survival in the per-protocol population

**
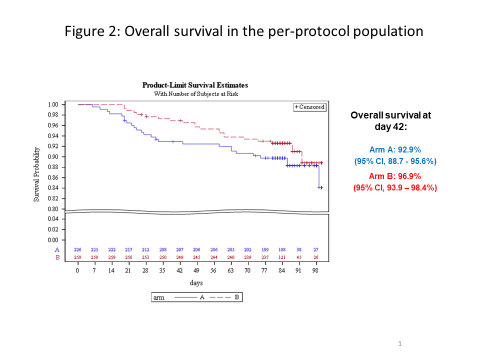
**

Figure S2 timing of the proven/probable IFD by arm.


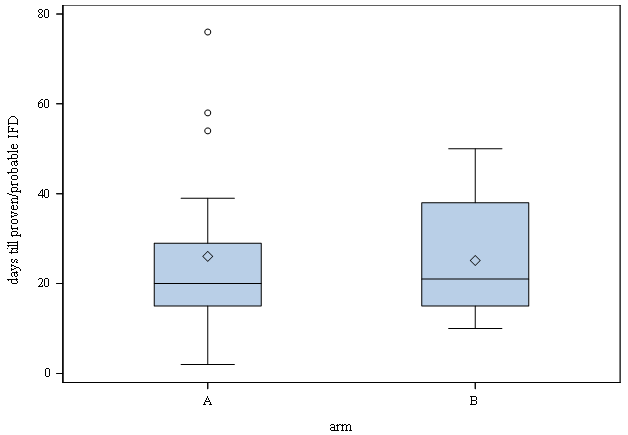


The 39 proven/probable IFDs occurred after median 21 days (IQR: 15 to 38 days) (min 2 days, max 76 days). In arm A, the 18 proven/probable IFDs occurred after median 20 days (IQR: 15 to 29 days). In arm B, the 21 proven/probable IFDs occurred after median 21 days (IQR: 15 to 38 days).
